# Did you even see that? visual sensory processing of single stimuli under different locomotor loads

**DOI:** 10.1371/journal.pone.0267896

**Published:** 2022-05-26

**Authors:** Julian Elias Reiser, Stefan Arnau, Gerhard Rinkenauer, Edmund Wascher

**Affiliations:** Leibniz Research Centre for Working Environment and Human Factors, Dortmund, Germany; Universita degli Studi di Roma La Sapienza, ITALY

## Abstract

Modern living and working environments are more and more interspersed with the concurrent execution of locomotion and sensory processing, most often in the visual domain. Many job profiles involve the presentation of visual information while walking, for example in warehouse logistics work, where a worker has to manage walking to the correct aisle to pick up a package while being presented with visual information over data-glasses concerning the next order. Similar use-cases can be found in manufacturing jobs, for example in car montage assembly lines where next steps are presented via augmented reality headsets while walking at a slow pace. Considering the overall scarcity of cognitive resources available to be deployed to either the cognitive or motor processes, task performance decrements were found when increasing load in either domain. Interestingly, the walking motion also had beneficial effects on peripheral contrast detection and the inhibition of visual stream information. Taking these findings into account, we conducted a study that comprised the detection of single visual targets (Landolt Cs) within a broad range of the visual field (-40° to +40° visual angle) while either standing, walking, or walking with concurrent perturbations. We used questionnaire (NASA-TLX), behavioral (response times and accuracy), and neurophysiological data (ERPs and ERSPs) to quantify the effects of cognitive-motor interference. The study was conducted in a Gait Real-time Analysis Interactive Laboratory (GRAIL), using a 180° projection screen and a swayable and tiltable dual-belt treadmill. Questionnaire and behavioral measures showed common patterns. We found increasing subjective physical workload and behavioral decrements with increasing stimulus eccentricity and motor complexity. Electrophysiological results also indicated decrements in stimulus processing with higher stimulus eccentricity and movement complexity (P3, Theta), but highlighted a beneficial role when walking without perturbations and processing more peripheral stimuli regarding earlier sensory components (N1pc/N2pc, N2). These findings suggest that walking without impediments can enhance the visual processing of peripheral information and therefore help with perceiving non-foveal sensory content. Also, our results could help with re-evaluating previous findings in the context of cognitive-motor interference, as increased motor complexity might not always impede cognitive processing and performance.

## Introduction

In everyday life, it has become common to be overstrained with visual information while in a state of active locomotion. Many job profiles involve the presentation of visual information while walking, for example in warehouse logistics work, where a worker has to manage walking to the correct aisle to pick up a package while being presented with visual information over data-glasses concerning the next order. Similar use-cases can be found in manufacturing jobs, for example in car montage assembly lines where next steps are presented via augmented reality headsets while walking at a slow pace. The information is not presented at the foveal point of vision as it would occlude important visual information of the upcoming pathway, but in the peripheral field of view. While stimuli presented in peripheral visual areas were found to go along with increased response times and decreased accuracy [1, 2], walking seems to enhance peripheral vision capabilities and equate these decremental effects [3, 4]. Yet, we do not know, if the interaction of processing peripheral, lateralized visual information and active locomotion imposes a high or low cognitive demand on the worker.

When thinking about processing visual stimuli and a concurrent walking task, one would generally expect to encounter a decrement in either or both task domains depending on task demands—a phenomenon called cognitive-motor interference (CMI; for a meta-analysis see [5]). Based on theories of limited resources, CMI should lead to overall resource depletion when two or more tasks with overall demands that exceed the subject’s resource capacities are executed concurrently [6–8]. This maladaptation between demands and resources might result in diminished behavioral performance, e.g. increased error rates (cognitive domain) and/or reduced gait speeds (motor domain). In the most severe case, reduced availability of attentional resources due to CMI could lead to a reduction in gait stability and result in a fall and succeeding injury [9, 10]. While many studies outline a detrimental effect of increased movement demands on cognitive tasks, some exceptions indicate either no differences in behavioral performance between stationary and locomotive states [11–13] or a performance increase under aerobic exercise compared to a resting state [14]. As research on the topic of CMI is ongoing, the impact of locomotion on a cognitive task is also strongly dependent on the intensity and difficulty of the motor task used—and therefore the demands of concurrent dual-tasking.

As walking and visual sensory processing are therefore tightly interconnected [3, 15], it is important to understand their relationship to one another and how different stages of locomotion load might affect underlying cognitive mechanisms. For an evaluation of such concurring resource demands, electrophysiological measurements have proven to give a detailed insight into the intricacies of cognitive resource distribution. Here, especially the EEG has been utilized due to its mobility, lightweight, sufficient spatial, and excellent temporal resolution [16, 17].

While there have been many electrophysiological studies that looked into the effects of concurrent locomotor and visual load, either in laboratory facilities [13, 18–22] or more naturalistic environments [23, 24], only a few studies dealt with the topic of the processing of peripheral visual information while walking [3, 4, 11]. While animal studies showed clear evidence that locomotion changed visual processing so that the suppression of peripheral visual information was decreased [25–28], studies on human subjects could not establish such a relation [3, 4, 29]. In one of these studies with humans, on the other hand, Benjamin, Wailes-Newson, Ma-Wyatt, Baker, and Wade [3] could show that early visual contrast processing was elevated in humans in the peripheral visual field. Using steady-state visual evoked potential paradigms, these results were shown for subjects that walked on a treadmill being presented with visual stimuli via a PC screen [3] or for subjects that walked in a sports hall with stimuli being presented using a head-mounted display [4].

To gather insights into the underpinnings of sensory and attentional processes, event-related potentials (ERPs) and spectral perturbations (ERSPs) of the EEG have been used over decades to investigate cognitive mechanisms. Even in motion and outside of regular laboratory facilities, these techniques can be used to deduct liable information to answer specific questions about cognitive processes with high temporal resolution (e.g. [30–32]). There are certain components of high interest when dealing with lateralized visual stimuli in a cognitive-motor interference paradigm.

The fronto-central N2 has been of interest in many concurrent cognitive-motor dual-task studies. In general, the component was shown to indicate inhibitory and executive functional processes, being strongly dependent on the activity of the prefrontal cortex [33–35]. Though previous laboratory studies demonstrated that N2 amplitude is increased with higher inhibitory task demands [33, 35], the overall result pattern of studies using mobile cognitive-motor dual tasks is less clear. No difference in N2 amplitude could be found in an auditory oddball task [36] or an auditory task-switch task [31] while participants stood, walked, or traversed an obstacle course, while De Sanctis, Butler, Malcolm, and Foxe [13] found a decrease in N2 amplitude for walking compared to standing in a visual go/nogo task.

Another measure that was placed in the context of central executive functioning and cognitive interference is frontal event-related Theta (4–7 Hz) power [37, 38]. Results highlighted that with increased cognitive control demands, stronger frontal Theta activation was exhibited [39–41]. In mobile EEG studies, there were decrements found in frontal Theta when walking or traversing an obstacle course compared to standing still while executing an oddball or task-switch paradigm in an outdoor environment [31, 36]. This finding might indicate a decrease in available cognitive resources with increased motor load whereas differences in cognitive task difficulty did not influence Theta amplitude. This, again, demonstrated the lack of overall shared resources in a CMI task when motor load increased.

The centro-parietal P3 ERP component was found to be indicative of several cognitive processes, most prominently as a correlate of cognitive resource deployment and processing capacity in dual-task paradigms [42]. It was found that a decrease of P3 amplitude and a higher latency of the P3 peak were accompanied by a behavioral decrement in the respective task. It has also been used several times in CMI mobile EEG studies as an indexing method of cognitive dual-tasking; many studies found a decrease in P3 amplitude during walking compared to a still state [18, 20, 30, 36, 43]. This decrease in P3 amplitude in mobile brain/body imaging (MoBI) settings was found to be due to the change of visual and inertial information while in motion and not due to motion artifacts or the act of locomotion itself [30]. While many studies indicate a decrease in P3 amplitude, individual investigations highlight no change [12] or even a beneficial impact of bodily activity (walking, cycling) on P3 amplitude [14]—therefore also on cognitive resources to be spent on the cognitive task.

The N1, meaning the first negative deflection after stimulus presentation, measured at posterior (p) sites contralateral (c) to the presented stimulus (hence N1pc) was found to indicate early sensory processing in the primary visual cortex related to the stimulus’ physical characteristics [44, 45]. This first negativity was observed to be increased with higher stimulus saliency [44]. In a following stimulus processing step, a second posterior and contralateral negativity (N2pc) was found to indicate the allocation of attentional resources to a given visual target. Several studies have found that this component was increased when selective attentional resources were needed to attend to a target in a group of distractors [44, 46, 47]. While there are no results regarding the N1pc in moving subjects, a recent study showed no significant difference between N2pc amplitudes comparing standing and freely walking subjects while identifying a visually presented target detection task [48].

To address the question of how peripheral visual information is processed while walking, we conducted a study in a controlled environment, using neurophysiological measures. By excluding environmental distractors as would be the case in out-of-the-lab experiments, we could make sure to evaluate cognitive processing of the presented stimuli [49], as environmental scenery could have hindered clear inferences about the intricacies of visual processing [31]. We used a tiltable and swayable treadmill, part of the Gait Real-Time Analysis Laboratory (GRAIL, Motek Medical, Utrecht, NL) to induce locomotion states of automatic, fixed speed walking and more complex locomotion with a fixed speed and timed, abrupt side-sways so that participants had to stabilize their gait. Participants were either standing, walking, or walking with perturbations while being presented with single Landolt rings at various eccentricities that demanded a manual response depending on their opening, constituting a CMI dual-task. Though treadmill walking does not allow for direct conclusions about CMI in the real-world [50], we could evaluate the effects of automatic and perturbed states of locomotion on early and later stages of cognitive processing. To our knowledge, there hasn’t been a study investigating the questions at hand.

As this study is one of the first of its kind, we had several expectations about possible results. We expected subjective workload ratings (NASA-TLX; [51]) to increase with increased motor load. Response times were hypothesized to increase with both motor complexity and stimulus eccentricity, whereas accuracies should decrease with increasing motor load and eccentricity [1, 2, 11, 29, 31]. Regarding neurophysiological measures, the N1pc/N2pc complex should be increased in amplitude with increasing attentional resources applied to the task—if available due to the dual-task approach used here. Following the findings of increased peripheral vision in regular, more or less automatic locomotion, we expect that N1pc/N2pc amplitudes should be increased in automatic walking as compared to standing or perturbed walking. Also, increasing stimulus eccentricity should result in lower saliency due to the human visual system. Because of this lower saliency, higher eccentricity should lead to a decrease in N1pc/N2pc amplitude. The frontal N2 amplitude, as well as midfrontal Theta power, both indicate the need for cognitive control [40]. We, therefore, expect these measures to be elevated for increased perceptual load (higher eccentricities) and to be decreased for the highest motor load conditions [31]. P3 amplitudes, on the other hand, should be diminished as a function of increased dual-task load whereas latencies should increase. Both increased motor complexity and eccentricity should lead to a reduction in overall available cognitive capacities, finding its expression in lower P3 amplitudes and higher latencies.

## Methods

### Participants

For this sample, 23 participants (12 female, 9 male) without any prior or present neurologic or psychiatric condition were recruited using online announcements and postings on bulletin boards at the Technical University Dortmund between early August and end of November 2020. All participants had no motor or gait impairment, had normal or corrected-to-normal vision, and were right-handed. Due to minor technical difficulties in the communication between devices, only 21 datasets were used for further analysis. The participants’ age ranged from 18 to 33 years (*M* = 24.29, *SE* = 0.70). Before the experiment, participants gave their informed written consent. Study participation was compensated with class credit or monetary reimbursement of 10 € per hour. The study was approved by the local ethics committee of the Leibniz Research Centre for Working Environment and Human Factors and was conducted in accordance with the Declaration of Helsinki. Also, safety measures were adhered to, thereby excluding individuals who have been in contact with Covid-19 patients and who either lived in or traveled to areas where Covid-19 infection rates were above a certain threshold.

### Apparatus

**Fig 1:**
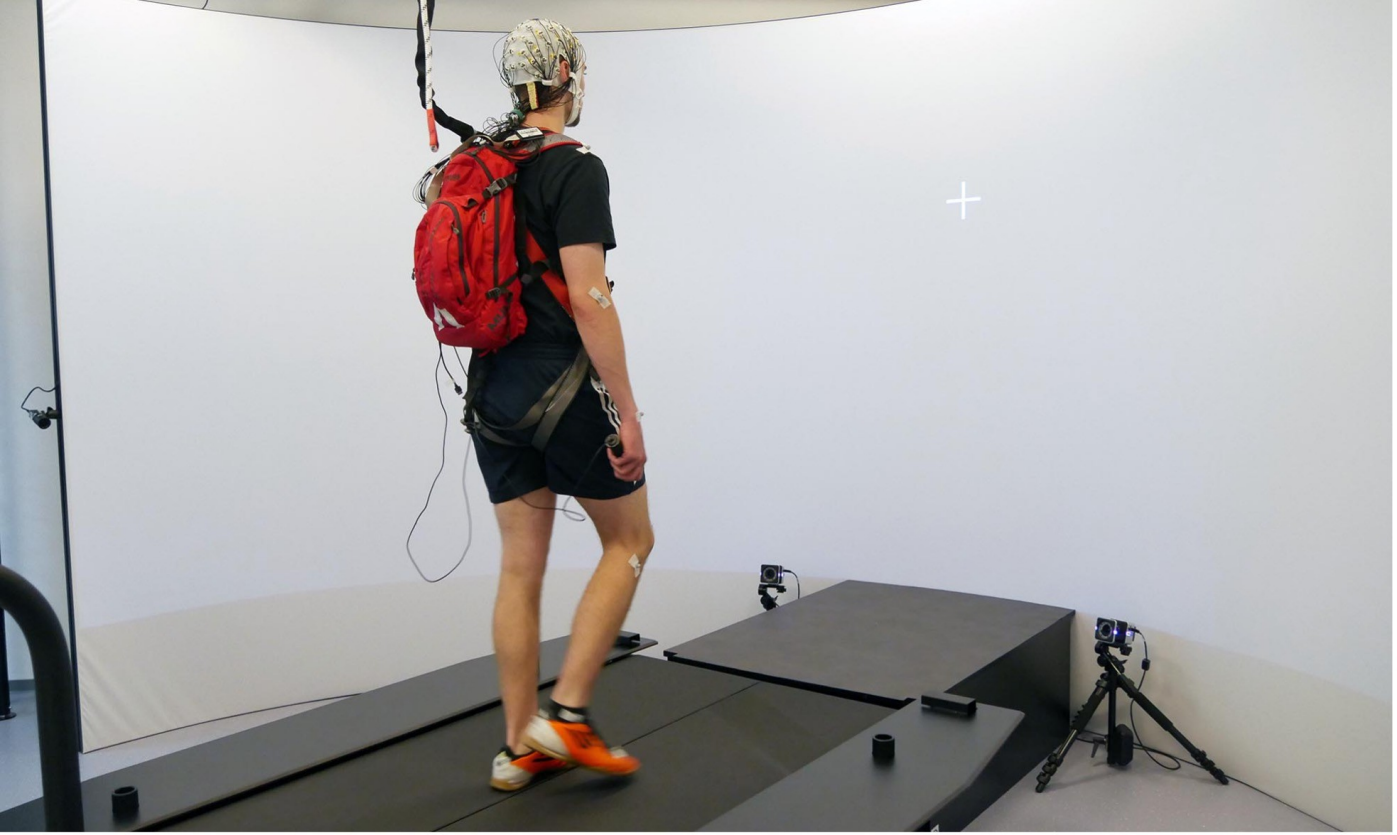
Participant performing the visual search task while walking on the GRAIL treadmill looking at the fixation cross. Stimuli were presented in the range from -40° to +40° degree visual angle and centered at eye level. In the perturbation blocks, the treadmill swayed randomly to the left or the right at the exact moment of stimulus presentation.

All data used for this study were recorded in the GRAIL facilities at the Leibniz Research Centre for Working Environment and Human Factors (Dortmund, Germany; Fig 1). The GRAIL consists of an instrumented dual-belt treadmill with two embedded force plates, a VICON motion capture system (VICON, Oxford, United Kingdom) using ten infrared cameras, a 180° projection screen in front of the treadmill, and four projectors for visual scene presentation on the screen and the frontal treadmill area. The treadmill platform was also able to be tilted forwards and backward, as well as to be moved vertically to induce mechanical perturbations. Visual stimulus presentation and treadmill operation were done using the integrated D-flow software (Motek Forcelink, Amsterdam, Netherlands). Participants wore a safety harness connected to the ceiling throughout the whole time of experimentation to prevent falls or injury.

To be able to synchronize visual stimulus onset timings and the ongoing EEG signal, an external trigger interface (Phidget Interface Kit, Phidgets Inc., Canada) was used to generate trigger signals whenever a stimulus was presented via the projectors. Then, to simultaneously record trigger signals and ongoing EEG data we used the wireless trigger set by Brain Products (Gilching, Germany). Therefore, we attached the wireless transmitter to the trigger interface and the wireless receiver to the sensor and trigger extension box (Brain Products GmbH) that was connected to the LiveAmp EEG amplifier (Brain Products GmbH) via a cable.

**Fig 2:**
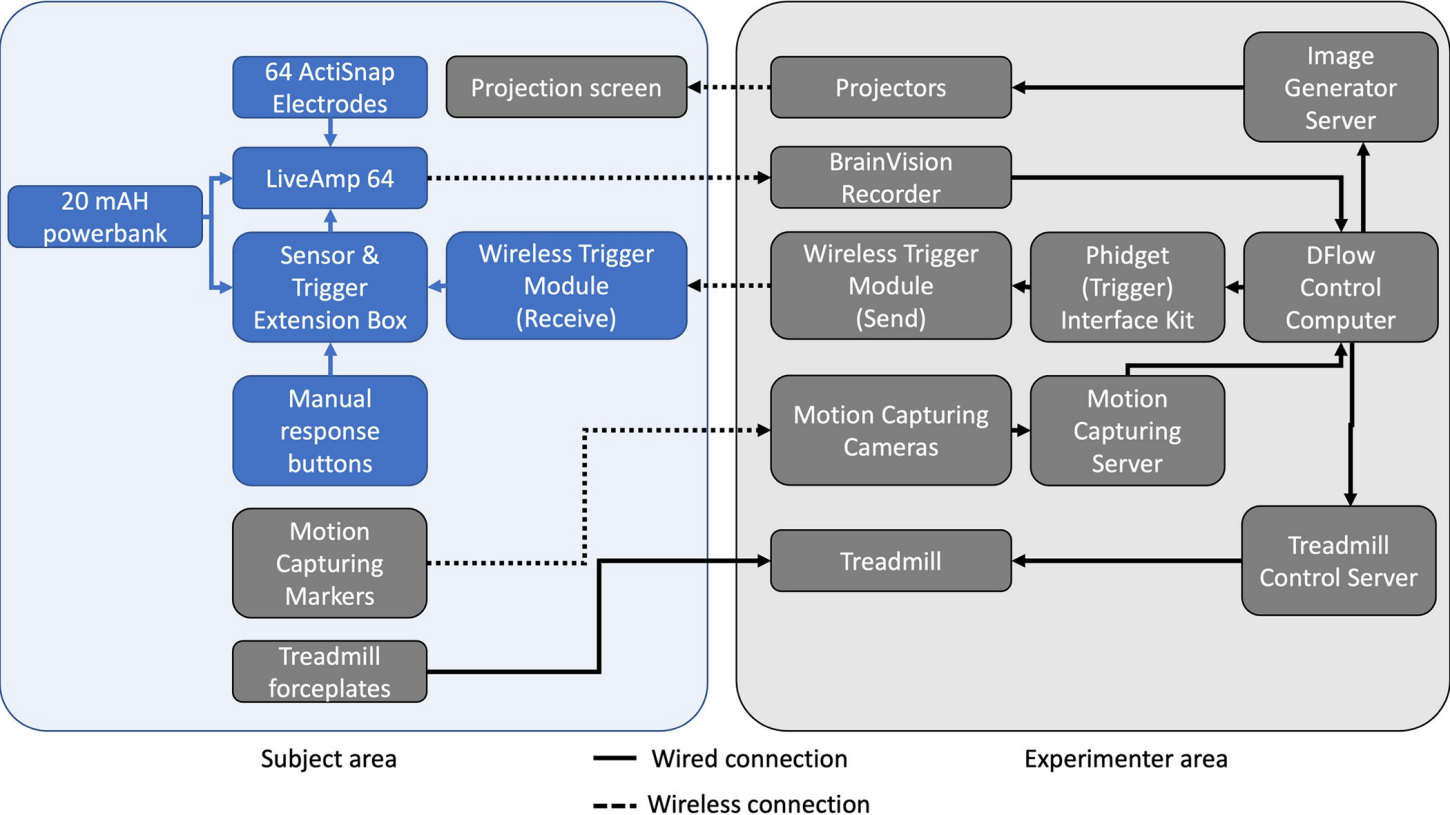
Schematic set-up of the experimental apparatus. The subject’s area includes the treadmill and projection screen portion of the GRAIL, the experimenter’s area is located on the control desk including a server rack in an adjacent room. Blue boxes indicate every device attached to or held by the participant, whereas black boxes involve all other elements of the set-up. Also, the dotted lines indicate wireless transmission of signals, solid lines symbolize wired connections.

Manual responses were made possible with the help of two custom-made tethered response buttons (made from bicycle handlebar grips and a button on top) one of which was held by the participant in each hand. These buttons were also connected to the extension box so that triggers were simultaneously recorded with the ongoing EEG signal. Both the extension box and the wireless trigger receiver, as well as portable power banks connected to the technical devices, were stored in a backpack worn by the participant throughout the experimental session. For a schematic depiction of the experimental set-up, please see Fig 2.

### Stimuli and task

All experimental blocks consisted of the concurrent execution of a locomotor and a visual cognitive task. Regarding the locomotor task, participants either stood on the treadmill, walked with a fixed speed of 1.2 meters per second, or walked with this fixed speed of 1.2 meters per second while their gait was perturbed. A perturbation consisted of an abrupt horizontal movement of the treadmill (lateral distance: 2,5 cm, time of the movement cycle: 800 ms), either to the right or to the left side, and was performed simultaneously to the visual stimulus onset. The side of perturbation was randomized.

**Fig 3:**
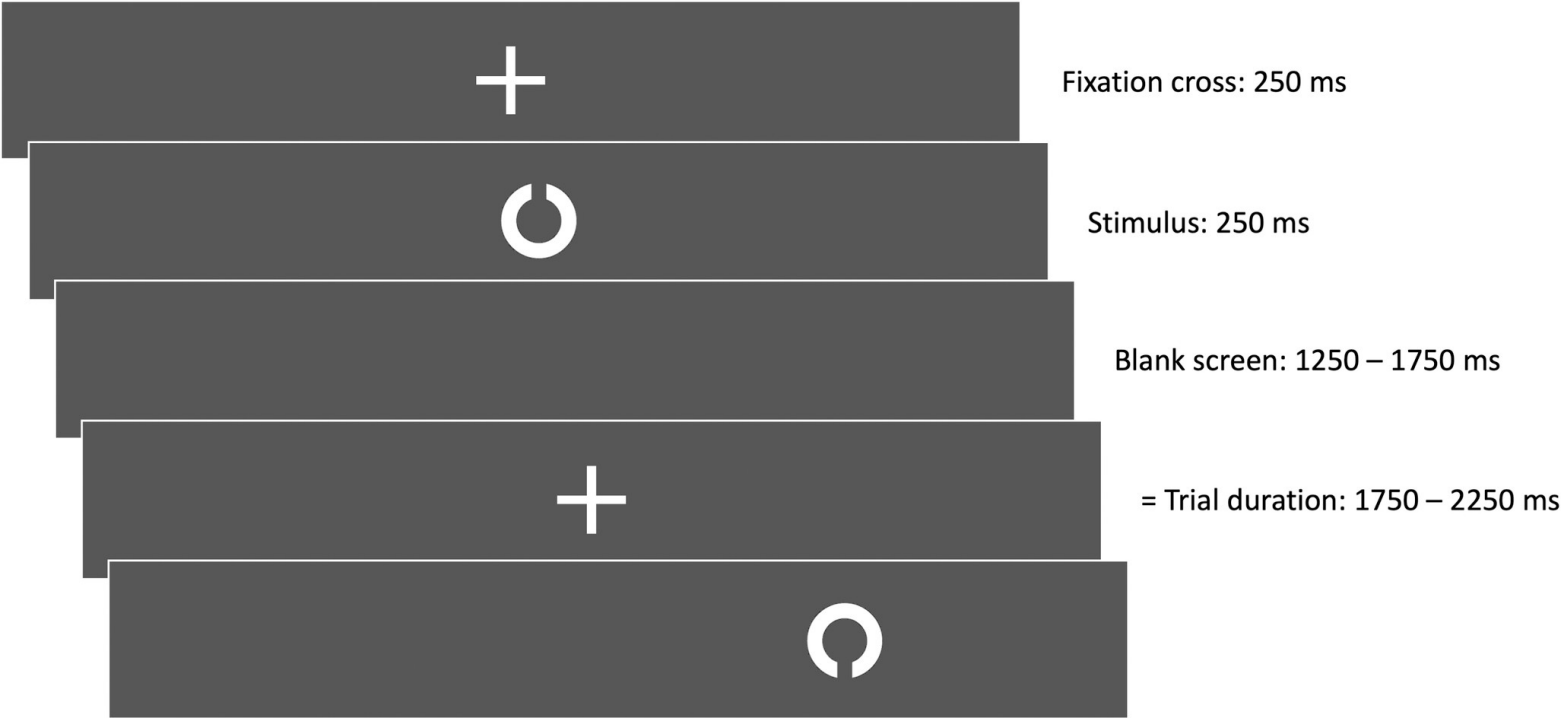
The course of a trial with one Landolt ring stimulus. The trial starts with a 250 ms fixation cross, being followed by the stimulus presentation for 250 ms. This stimulus is presented between 0° and 40° visual angle. Next, there is an empty screen before the next fixation cross presentation. Responding manually is possible throughout the whole trial.

In terms of the visual task, participants were presented with a single Landolt-ring with an opening at the top or on the bottom. These rings were constructed according to the EN ISO 8596 norm with a 15 cm diameter and a 3 cm opening. Participants had the task to indicate to which side the target Landolt ring was opened via a left- or right-hand button press. To prevent bias, hand assignments to the openings were balanced throughout the sample. A trial started with the presentation of the fixation cross for 250 ms, followed by the Landolt stimulus for 250 ms. The manual response window had a mean length of 1500 ms (see Fig 3). The overall interstimulus interval was jittered by +/- 250 ms so that participants could not align their gait to the stimulus presentation. The background of the projection was not completely black, but 20% grayscale (relative luminance from text to background: 1 : 18.423) to decrease the visibility of projector light cross-bleed where the projection fields of the individual projectors met.

For all measures that include eccentricity values, the continuously presented stimuli were binned into three categories of eccentricity: foveal (0–4° visual angle), extrafoveal (4–18° visual angle), and peripheral (18–40° visual angle). This leaves us with a 3x3 factorial design consisting of three levels of the motor task (standing, walking, walking with perturbations) and three levels of the visual task (central, extrafoveal, peripheral stimulus). Each block, participants were presented with 300 trials per condition. All blocks took approximately 10 minutes, resulting in an experimentation time of 60 minutes. The sequence of the resulting six tasks was pseudo-randomized in a block-wise manner using a Latin square design to prevent time-on-task effects and task sequence biases.

### Procedure

Participants arrived at 9:00 am at the institute. Upon arrival, the participant’s temperature was checked before entering the institute building and they were handed a COVID19 questionnaire to reduce the risk of infection. After they filled out the form, participants were escorted to the GRAIL laboratory where they read the experiment’s information sheet, gave their informed consent, and filled out a demographics questionnaire. To avoid faulty motion tracking reflections, the participant was escorted onto the treadmill to check for reflective surfaces on their clothes which were then covered with tape.

Subsequently, participants were escorted back to the operator space and were fitted with a 64-electrode cap (actiCap Snap, Brain Products GmbH, Germany) equipped with slim, active Ag/Cl electrodes (actiCap slim, Brain Products GmbH, Germany). The electrodes were filled with electrolyte gel to reach an impedance equal to or below 10 kΩ. Followingly, participants put on the backpack enclosing the trigger extension and the trigger WiFi module. The two LiveAmp amplifiers (LiveAmp 64, Brain Products GmbH, Germany) were then stored in the backpack and all components were connected to off-the-shelve power banks to avoid running out of battery throughout the experiment. Next, 26 reflective body markers were taped to the specific landmarks of the participant to later measure full-body motion using the laboratory’s embedded VICON system.

After this 60-75-minute preparation process, the experimenter escorted the participant to the treadmill, strapped the safety harness to the ceiling security cord, and started the VICON body model calibration procedure (raising arms in front of their body, walking on the spot, walking forwards, walking backward). After calibration, the EEG measurement was started and a standardized verbal explanation of the tasks was given by the experimenter; also, the fixation cross and stimulus presentation height was adjusted to match the participants’ eye level. In the following step, 3-minute baseline blocks were recorded for each locomotive condition (standing, walking, perturbed walking) before starting with experimental dual-tasks. After each block, participants were asked to fill out a NASA-TLX questionnaire [51] to assess subjective workload and encouraged to take a rest. Once the experiment was over, the equipment was removed and participants were escorted to the bathroom, where they could change and wash their hair.

### Questionnaire and behavioral data

For the assessment of the subjective workload, a German translation of the NASA-TLX was used. For analysis, all six individual subscales (cognitive workload, physical workload, time demands, performance, effort, frustration) were analyzed. Response times were calculated as the latency between stimulus onset and correct button press. Also, to be categorized as a correct answer, the button press latency had to be committed before the next cue onset. Accuracy was computed as the ratio of correctly answered trials and all trials.

### Electrophysiological data acquisition

EEG data were acquired with 64 electrodes in a standard 10–20-system montage. FCz was used as the online reference and AFz served as the ground electrode. After fitting a tight cap with circumferences ranging from 54 to 60 cm equipped over the participant’s head, the electrodes were filled with conductive gel. It was ensured that electrode cables did not cross each other and had no clearance to sway around to prevent electric motion artifacts. All cables were routed through loopholes on the sides of the cap into the mobile LiveAmp 64 EEG amplifier which was placed in the backpack carried by the participants. Data were recorded with a sampling rate of 500 Hz and a bit depth of 24 bit directly onto a micro-SD card inserted into both connected amplifiers. At the same time, the online data stream which was received from the amplifiers’ Bluetooth connection was monitored from the lab’s D-Flow controller computer using the Brainvision Recorder software (Brain Products GmbH). Upon completion of the experiment, the EEG data were transferred from the SD card to the laptop using the LiveAmp File Converter software (Brain Products GmbH).

### Data processing

The recorded datasets were analyzed using custom MATLAB scripts (version 2020b, Massachusetts, The MathWorks Inc.) and functions of the EEGLab toolbox (Delorme & Makeig, 2004). Continuous data were band-pass filtered between 0.5Hz and 30Hz using a fourth-order IIR Butterworth filter with DC offset removal. Then, channels were rejected based on their kurtosis (threshold 8 SD) and probability (threshold 5 SD) using EEGLab’s pop_rejchan function (number of rejected channels: M = 3.52; SD = 1.91). After rejection, missing channels were interpolated so that all channels could be re-referenced to average reference. A separate independent component analysis (ICA) dataset was created by filtering the continuous data between 1 and 30 Hz using the EEGLab butterworth filter (filter order 4) and downsampling the filtered signal to 125 Hz to facilitate ICA decomposition. Then, both the EEG- and the ICA-dataset were epoched to -800 ms to +2400 ms relative to stimulus onset. A baseline in the time-range 200 ms before cue-onset ranging from -450 ms to -250 ms before the stimulus was subtracted from the data of all trials. Before ICA decomposition, epochs were rejected using EEGLab’s built-in epoch-rejection function (voltage threshold: 500 μV, probability threshold: 5 SD, maximum percent of total trials to reject per iteration: 10%; rejected epochs: M = 266.10; SD = 149.11). An ICA with a PCA to avoid rank-deficiency (59 –number of rejected channels) was performed on the ICA-dataset and the independent component (IC) spatial filters were copied to the 500 Hz dataset. The rationale behind interpolating before ICA and using a PCA afterward was as follows: The topographical projections of the ICs can be biased if excluded channels are not distributed evenly across the scalp. This, in return, might impede the capability of the IC classifier ICLabel to detect artifact ICs reliably. For this reason, we interpolated excluded channels before computing the ICA. The interpolation of missing channels, however, does not add further information to the data. Therefore, the resulting data might be rank-deficient which can reduce the quality of source separation. Data compression by PCA before running the ICA can guarantee full-rank data (for further information see Makoto’s preprocessing pipeline: https://sccn.ucsd.edu/wiki/Makoto%27s_preprocessing_pipeline).

To clean the data further, artifact-related ICs were excluded by using the ICLabel classifier plugin for EEGLab (Pion-Tonachini, Kreutz-Delgado, & Makeig, 2019). All components that were classified as having a probability of less than 50% to be a “Brain IC” were excluded from the EEG dataset. As ICLabel classifiers were trained on laboratory data, all classification solutions were checked by the corresponding author before advancing with the rest of the analysis. Also, every IC flagged for exclusion was examined individually (rejected ICs: M = 33.29; SD = 5.92).

### EEG parametrization and analysis

#### ERPs

The preprocessed data were averaged for each channel and each experimental condition. To quantify amplitudes and latencies in each time window, we used 50% fractional area latency (FAL) and area amplitude (AA) for each chosen component. FAL was calculated by searching for the crossings of the x-axis within a certain time window in the individual ERPs which served as start and endpoints of the FA detection (if no x-axis crossings were detected, the start- and/or the endpoint of the time window were used; for positive components only positive values within the time window were considered, for negative components we only took negative values). Within this time window, the overall area under the curve was estimated as the cumulative sum of values over/under the x-axis dependent on the polarity of the component. After 50% of the cumulative sum was reached, the corresponding time code was quantified as the FAL. For AA quantification, we took the cumulative sum of the time window’s amplitudes.

In the case of asymmetries, the area detection was applied to the difference wave of contralateral—ipsilateral activation. This fractional area quantification was applied to each subject for each condition. To avoid subject-specific bias, all data were jack-knifed before this procedure. After AA / FAL quantification, a correction was applied for all participants for each condition due to the jack-knifed data [(*N* * FA_condition_)—((*N*—1) * FA_subject,condition_].

Using this approach, we quantified the N2 component at a frontal patch (Fz, FC1, FC2, Cz) in the time window between 250–500 ms after stimulus onset (GA peak: 386 ms). Also, the P3 was quantified at Pz in the time window between 250–600 ms after stimulus presentation (GA peak: 400 ms). To investigate lateralized visual processing, we quantified the N1pc/N2pc complex as the difference wave between contralateral minus ipsilateral activity measured at sites PO7 and PO8 related to stimulus position. Here, we defined time windows to investigate the N1pc between 130–250 ms (GA peak: 198 ms) and the N2pc between 230–340 ms after stimulus presentation (GA peak: 256 ms). Lateralized activity, meaning the N1pc/N2pc complex, was not calculated using stimuli from the foveal condition since a lateralized presentation of stimuli is needed to be able to compute said components.

#### ERSPs

Power values were extracted by wavelet convolution of each correct trial. Complex Morlet wavelets (defined as complex sine waves tapered by a Gaussian) were created by using 28 logarithmically spaced frequencies between 3 and 30 Hz with a full width half maximum in the temporal domain ranging from 360 to 100 ms. Power estimates for each time-frequency point were obtained by averaging the squared magnitude of the complex convolution result across trials. ERSPs were decibel-normalized within each experimental condition using a frequency-specific baseline ranging from −650 ms to −450 ms prior to stimulus onset. Theta-range activity was calculated as the mean of all frequencies between 4 Hz and 7 Hz. Again, the data were jack-knifed and quantified using the FAL/PA approach explained above. Here, we looked into Theta power dB at a frontal patch (Fz, FC1, FC2, Cz) in the time window between 180–300 ms after stimulus presentation (GA peak: 246 ms).

### Statistical analysis

Statistical analyses were carried out using Matlab’s built-in repeated measures ANOVA (fitrm, ranova) and pairwise t-Test. The general level of significance was set to 0.05. Whenever a sphericity correction was necessary, the p-value was Greenhouse–Geisser corrected (indicated by p_GG_). Also, to account for family-wise error accumulation, significances within each ANOVA were corrected for false discovery rate (FDR) as described by Cramer and colleagues [52]. Consecutive post-hoc tests were carried out using Matlab’s built-in pairwise paired t-test function. Post-hoc test probabilities were also corrected for FDR. In cases of FDR correction, adjusted critical p-values (p_crit_) are provided. Effect sizes for all tests are reported as adjusted partial eta squared following the article by Mordkoff [53]. Since this study follows a rather exploratory approach towards mobile electrophysiological recordings, also marginal effects (0.05 < p < 0.10) were reported and discussed.

## Results

### Questionnaires

**Fig 4:**
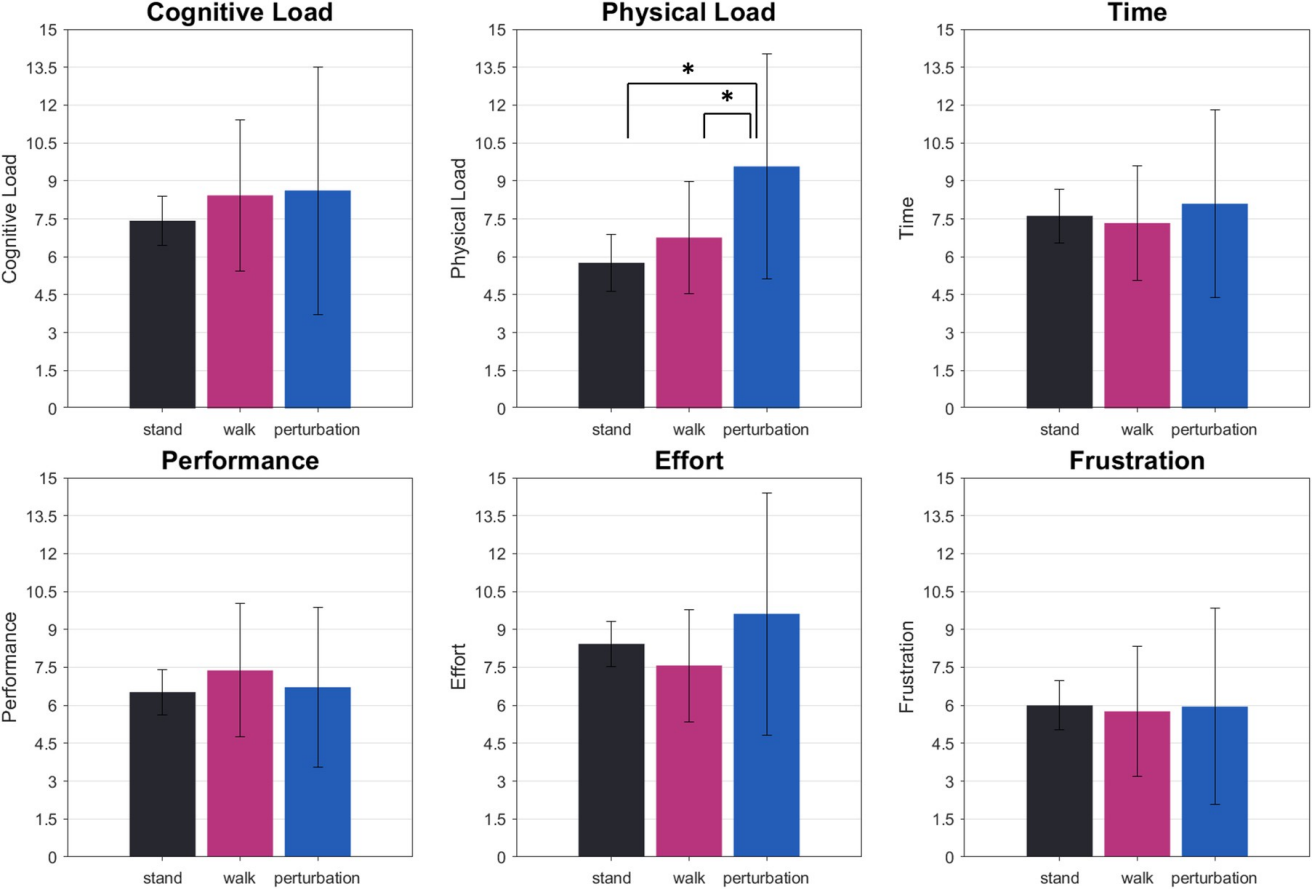
Means of all NASA-TLX subdimensions. The error bars indicate the standard error of the mean. Here, it is evident that only in the physical dimension subjective ratings of perceived workload differ significantly. There was no significant difference in the overall rating or the subdomains of the NASA-TLX besides for physical load. Here, perturbed walking was estimated to induce significantly higher subjective load than standing or regular treadmill walking. Significant differences (p < .05) are indicated by an asterisk.

Looking at the NASA-TLX subdimensions, only perceived physical load differed between motor load conditions, F(2,40) = 6.36, p_GG_ = .010, p_crit_ = .050, eta = 0.20. Here, standing (M = 5.76, SEM = 1.13), and walking (M = 6.76, SEM = 0.84) were rated to induce less subjective workload compared to perturbed walking (M = 9.57, SEM = 0.97; t(20) = -2.98). All other dimensions did not change with movement complexity, see Fig 4.

### Behavioral measures

**Fig 5:**
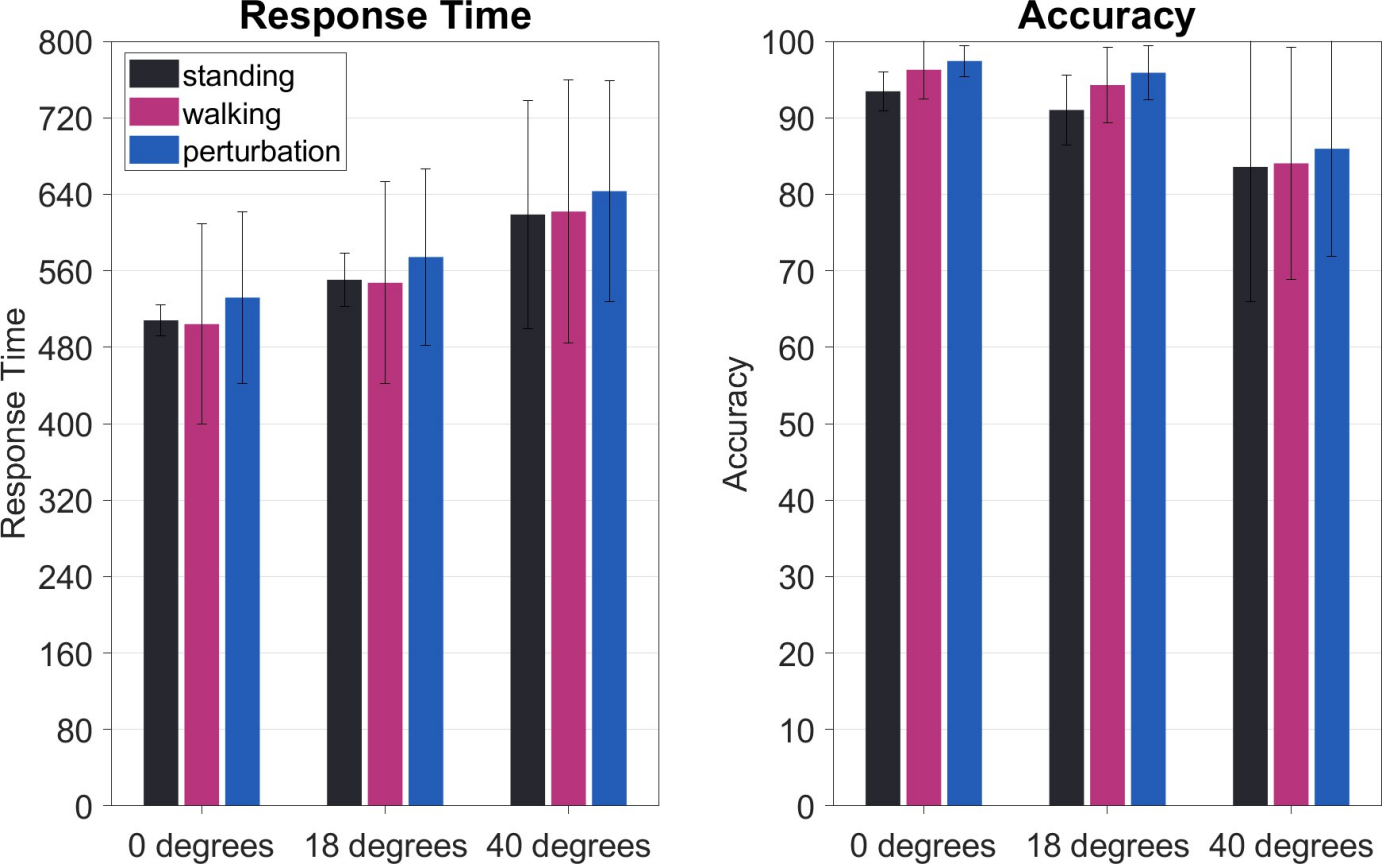
Response times in ms and accuracy in % correct answers. The error bars indicate the standard error of the mean. Considering response times, there was a significant difference between standing as well as regular walking as compared to perturbed walking. Also, response times increased significantly with increasing eccentricity. Regarding accuracy, only stimulus eccentricity significantly impaired responding correctly, whereas movement conditions did not influence response correctness.

Response times were influenced significantly by stimulus eccentricity. Marginally significant differences were also evident for motor complexity conditions. When having a look at the main effect of stimulus eccentricity, F(2,40) = 42.09, p_GG_ = < .001, p_crit_ = .050, $\eta_{part}^{2}$ = 0.66, response times increased with increasing degree of visual angle. As can be seen in Fig 5, response times increased significantly from foveal (M = 514.70; SEM = 18.49) to extrafoveal (M = 557.45; SEM = 19.42), t(20) = -8.46, p < .001, p_crit_ = 0.05, $\eta_{part}^{2}$ = 0.77, and from extrafoveal to peripheral stimulation (M = 628.01; SEM = 26.03), t(20) = -5.01, p < .001, p_crit_ = 0.17, $\eta_{part}^{2}$ = 0.53. Also, foveal and peripheral stimuli differed significantly, t(20) = -7.27, p < .001, p_crit_ = .033, $\eta_{part}^{2}$ = 0.71.

When looking at post-hoc differences of the main effect of movement complexity, F(2,40) = 3.49, p_GG_ = .041, p_crit_ = .033, $\eta_{part}^{2}$ = 0.11, there was a significant difference regarding perturbed walking (M = 583.17; SEM = 20.43) and standing (M = 559.14; SEM = 19.01), t(20) = -2.25, p = .04, p_crit_ = .05, $\eta_{part}^{2}$ = 0.16, and a marginal, but insignificant difference between perturbed walking and automatic walking (M = 557.85; SEM = 24.06), t(20) = -2.21, p = .04, p_crit_ = .033, $\eta_{part}^{2}$ = 0.16. Here, perturbations during walking seemed to increase response times whereas there was no difference between standing and walking regularly.

Accuracy, on the other hand, was not influenced by motor load, F(2,40) = 1.66, p_GG_ = .213, p_crit_ = .033, $\eta_{part}^{2}$ = 0.03, but only by stimulus eccentricity, F(2,40) = 13.92, p_GG_ = .001, p_crit_ = .050, $\eta_{part}^{2}$ = 0.38. Here, one could find that the highest eccentricity of 40° visual angle (M = .85; SEM = .03), had an impairing effect on accuracy when compared to foveal (M = .96; SEM = .01), t(20) = 3.93, p = .001, p_crit_ = .05, $\eta_{part}^{2}$ = 0.41, and extrafoveal stimulus presentation (M = .94; SEM = .01), t(20) = 3.49, p < .01, p_crit_ = .017, $\eta_{part}^{2}$ = 0.35. Differences between foveal and extrafoveal stimuli also reached significance, t(20) = 3.63, p < .01, p_crit_ = .033, $\eta_{part}^{2}$ = 0.37.

### Electrophysiological measures

#### Midline components: Frontal N2, frontal Theta, and parietal P3

**Fig 6:**
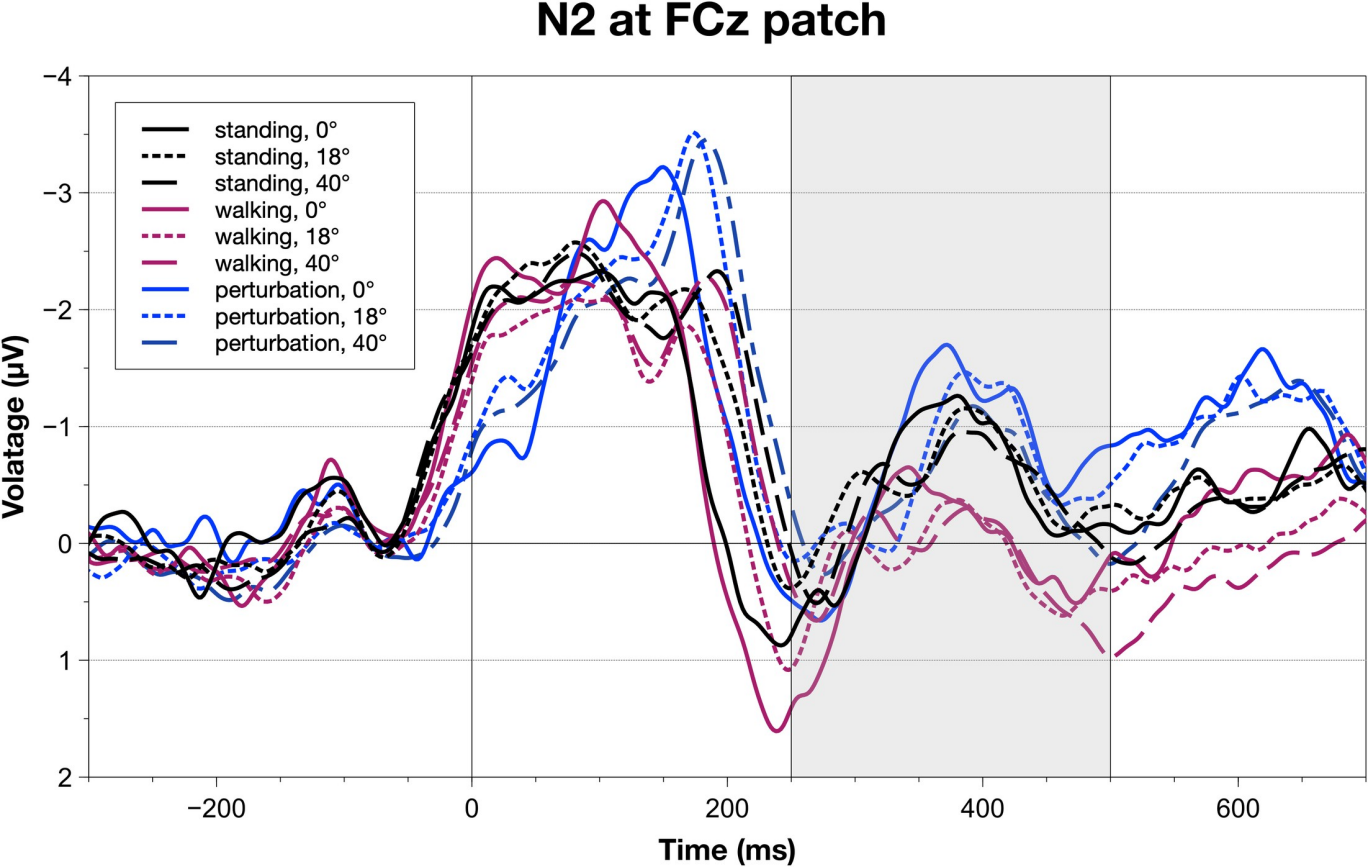
Grand average waveforms for all conditions calculated for an FCz patch (Fz, FC1, FC2, Cz). The grey rectangle indicates the area amplitude / fractional area latency window to parameterize the N2 component. Significant area amplitude main effects were found for both movement complexity and stimulus eccentricity. Fractional area latencies did not indicate any significant differences.

ERP and ERSP components recorded over the midline scalp surface indicated distinct processing patterns of foveal and lateralized stimuli in a CMI MoBI task. N2 area amplitudes (AA) indicated a significant main effect of movement complexity, F(2,40) = 7.12, p_GG_ = .003, p_crit_ = .050, $\eta_{part}^{2}$ = 0.23, and a marginal effect for stimulus eccentricity, F(2,40) = 3.96, p_GG_ = .044, p_crit_ = .033, $\eta_{part}^{2}$ = 0.12. Having a look at post-hoc differences, one can see that, interestingly, both standing (M = 80.47, SEM = 27.56) and perturbed walking (M = 83.63, SEM = 22.77) differed significantly from walking (M = 25.21, SEM = 17.64; standing vs. walking: t(20) = 3.14, p = .005, pcrit = 0.03, $\eta_{part}^{2}$ = 0.30; standing vs. perturbed walking: t(20) = -3.91, p .001, p_crit_ = 0.05, $\eta_{part}^{2}$ = 0.40). Regarding specific differences between stimulus eccentricities, it is evident that both extrafoveal (M = 66.36, SEM = 20.71) and peripheral stimuli (M = 43.50, SEM = 20.12) differed significantly from foveal ones (M = 79.46, SEM = 24.88; foveal vs. extrafoveal: t(20) = 1.16, p = .258, p_crit_ = 0.02, $\eta_{part}^{2}$ = 0.02; foveal vs. peripheral: t(20) = 2.28, p = .034, p_crit_ = 0.05, $\eta_{part}^{2}$ = 0.17). For a depiction of the grand averages, see Fig 6.

**Fig 7:**
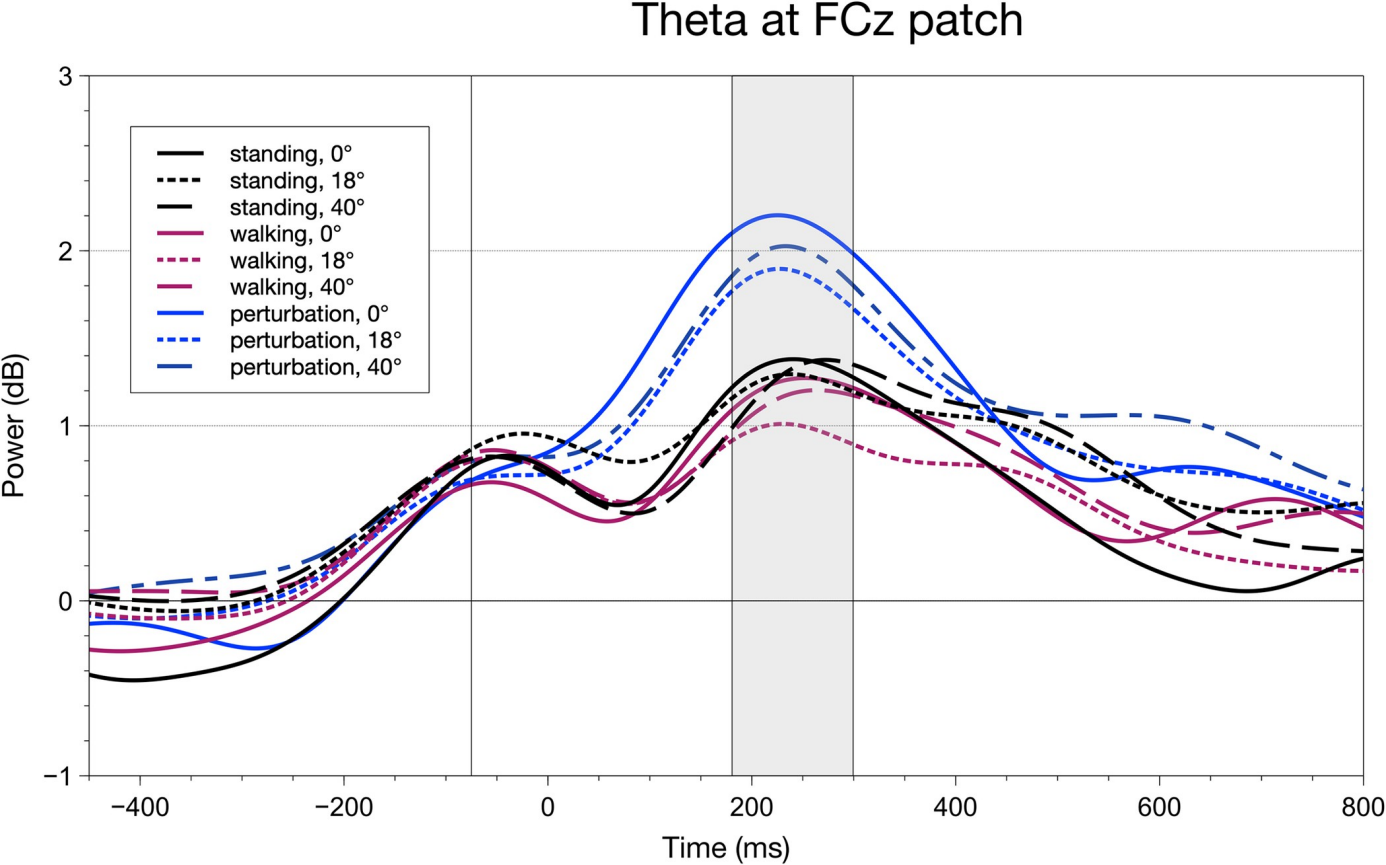
Grand average waveforms for all conditions calculated for an FCz patch (Fz, FC1, FC2, Cz). The grey rectangle indicates the area amplitude/latency window to parameterize Theta power. A significant area amplitude main effect was only found for movement complexity. Fractional area latencies did not indicate any significant differences.

A different pattern can be observed with fronto-central Theta power. Here, only movement complexity influenced the measure, F(2,40) = 12.89, p_GG_ = < .001, p_crit_ = .050, $\eta_{part}^{2}$ = 0.36). Interestingly, as opposed to N2 amplitudes, we could find significant differences not between walking and both other conditions, but between perturbed walking (M = 120.38, SEM = 19.66) and the other two, namely standing (M = 78.37, SEM = 19.59) and automatic walking (M = 67.90, SEM = 20.92). Here, we found a larger amplitude difference between automatic and perturbed walking, t(20) = -4.75, p < .001, p_crit_ = 0.05, $\eta_{part}^{2}$ = 0.51, when compared to standing and perturbed walking, t(20) = -4.10, p = .001, p_crit_ = 0.03, $\eta_{part}^{2}$ = 0.43. For a depiction of the grand averages, see Fig 7.

**Fig 8:**
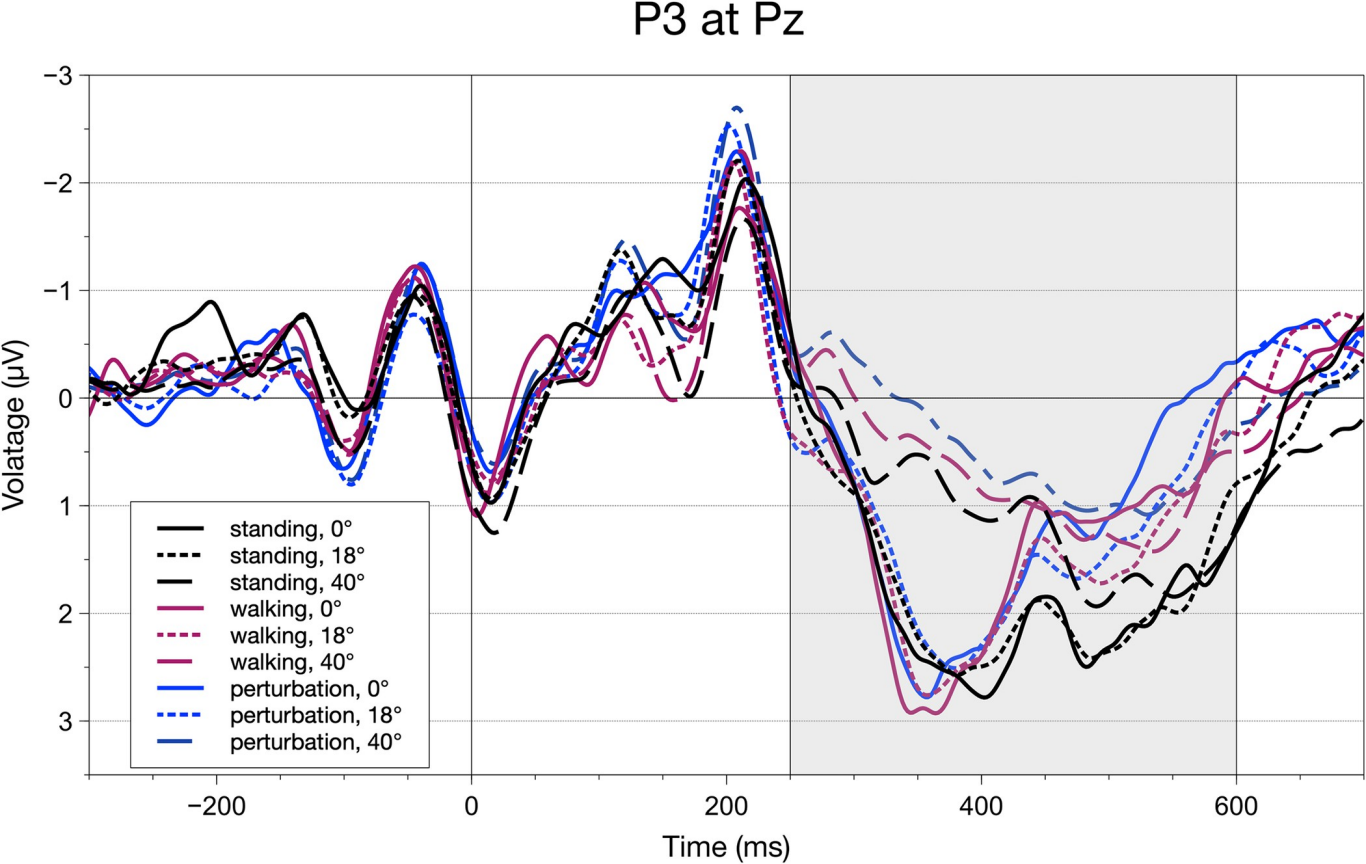
Grand average waveforms for all conditions calculated for electrode Pz. The grey rectangle indicates the area amplitude/latency window to parameterize the P3 component. Significant area amplitude main effects were found for both movement complexity and stimulus eccentricity. Fractional area latencies indicated significant differences for both main effects as well as the interaction.

When looking at P3 AA and fractional area latencies (FAL), one could see distinct effects of movement complexity and stimulus eccentricity. There were main effects regarding P3 amplitude for both movement complexity, F(2,40) = 12.17, p_GG_ = < .001, p_crit_ = .033, $\eta_{part}^{2}$ = 0.35, and stimulus eccentricity, F(2,40) = 16.20, p_GG_ = < .001, p_crit_ = .050, $\eta_{part}^{2}$ = 0.42. Looking at movement complexity conditions, standing (M = 273.75; SEM = 28.32) differed significantly from both regular walking (M = 205.65; SEM = 28.24), t(20) = 3.15, p = .01, p_crit_ = 0.033, $\eta_{part}^{2}$ = 0.30, and perturbed walking (M = 175.37; SEM = 26.99), t(20) = 4.85, p < .001, p_crit_ = 0.05, $\eta_{part}^{2}$ = 0.52. For eccentricity conditions, we could find significant differences between foveal (M = 251.60; SEM = 28.60) and peripheral stimuli (M = 138.00; SEM = 28.23), t(20) = 3.77, p = .001, p_crit_ = 0.033, $\eta_{part}^{2}$ = 0.39, as well as extrafoveal (M = 266.17; SEM = 29.98) and peripheral stimuli, t(20) = 6.10, p < .001, p_crit_ = 0.05, $\eta_{part}^{2}$ = 0.63. For a depiction of the grand averages, see Fig 8.

While rmANOVAs of fronto-central N2 and Theta FAL did not indicate any effects, P3 latencies showed an even more differentiated picture of results than P3 amplitudes. Here, both main effects of movement complexity, F(2,40) = 12.17, p_GG_ = < .001, p_crit_ = .033, $\eta_{part}^{2}$ = 0.35, and stimulus eccentricity, F(2,40) = 16.20, p_GG_ = < .001, p_crit_ = .050, $\eta_{part}^{2}$ = 0.42, were significant. Concerning different movement complexity conditions, there were significantly increased latencies for standing (M = 457.99; SEM = 10.90) compared to regular walking (M = 429.59; SEM = 9.26), t(20) = 3.26, p < .01, p_crit_ = 0.033, $\eta_{part}^{2}$ = 0.31, as well as perturbed walking (M = 431.70; SEM = 8.17), t(20) = 3.80, p = .001, p_crit_ = 0.05, $\eta_{part}^{2}$ = 0.39. Comparable to P3 amplitude results, latencies were significantly increased for peripheral stimuli (M = 489.61; SEM = 12.06) in comparison to foveal ones (M = 408.73; SEM = 8.91), t(20) = -6.27, p < .001, p_crit_ = 0.05, $\eta_{part}^{2}$ = 0.65, and extrafoveal stimuli (M = 420.64; SEM = 9.57), t(20) = -5.94, p < .001, p_crit_ = 0.033, $\eta_{part}^{2}$ = 0.62.

Also, we found an interaction effect, F(4,80) = 5.07, p_GG_ = < .01, p_crit_ = .017, $\eta_{part}^{2}$ = 0.35, that gave insights into significant differences of movement conditions within the different degrees of stimulus eccentricity (see Table 1 for all pairwise comparisons). Most interestingly, we see that while foveal stimuli were presented, only standing (M = 439.78; SEM = 13.10) went along with increased latencies compared to regular (M = 393.90; SEM = 11.06) and perturbed walking (M = 392.52; SEM = 8.62). This effect also showed for extrafoveal stimuli, where standing (M = 442.35; SEM = 12.30), had increased latencies in comparison to regular (M = 409.68; SEM = 11.03) and perturbed walking (M = 410.80; SEM = 9.58). This pattern only vanished when peripheral stimuli were presented while standing (M = 491.84; SEM = 13.47), walking (M = 485.21; SEM = 12.84), and perturbed walking (M = 491.77.; SEM = 13.79) eliciting approximately similar ERP morphologies.

**Table 1:** P3 fractional area latency post-hoc comparisons using paired-sample t-tests with FDR correction.

Comparison	t	df	p	p_crit_	$\eta_{part}^{2}$
0° stand vs. walk	3.79	20	.001	.044	.39
0° stand vs. perturbation	4.06	20	< .001	.05	.42
0° walk vs. perturbation	0.13	20	.90	.017	-0.5
18° stand vs. walk	2.96	20	.01	.033	.27
18° stand vs. perturbation	3.71	20	< .01	.039	.38
18° walk vs. perturbation	-0.13	20	.90	.011	-.05
40° stand vs. walk	0.73	20	.48	.028	-.02
40° stand vs. perturbation	0.01	20	.99	.001	-.05
40° walk vs. perturbation	-0.56	20	.58	.022	-.03

Note: Using paired sample t-tests, we compared mean differences between motor complexity conditions within each stimulus eccentricity condition separately. A comparison is significant when the p-value is less than the p_crit_-value. The effect size was calculated as partial eta-squared.

#### Lateralized components: Occipital N1pc/N2pc

**Fig 9:**
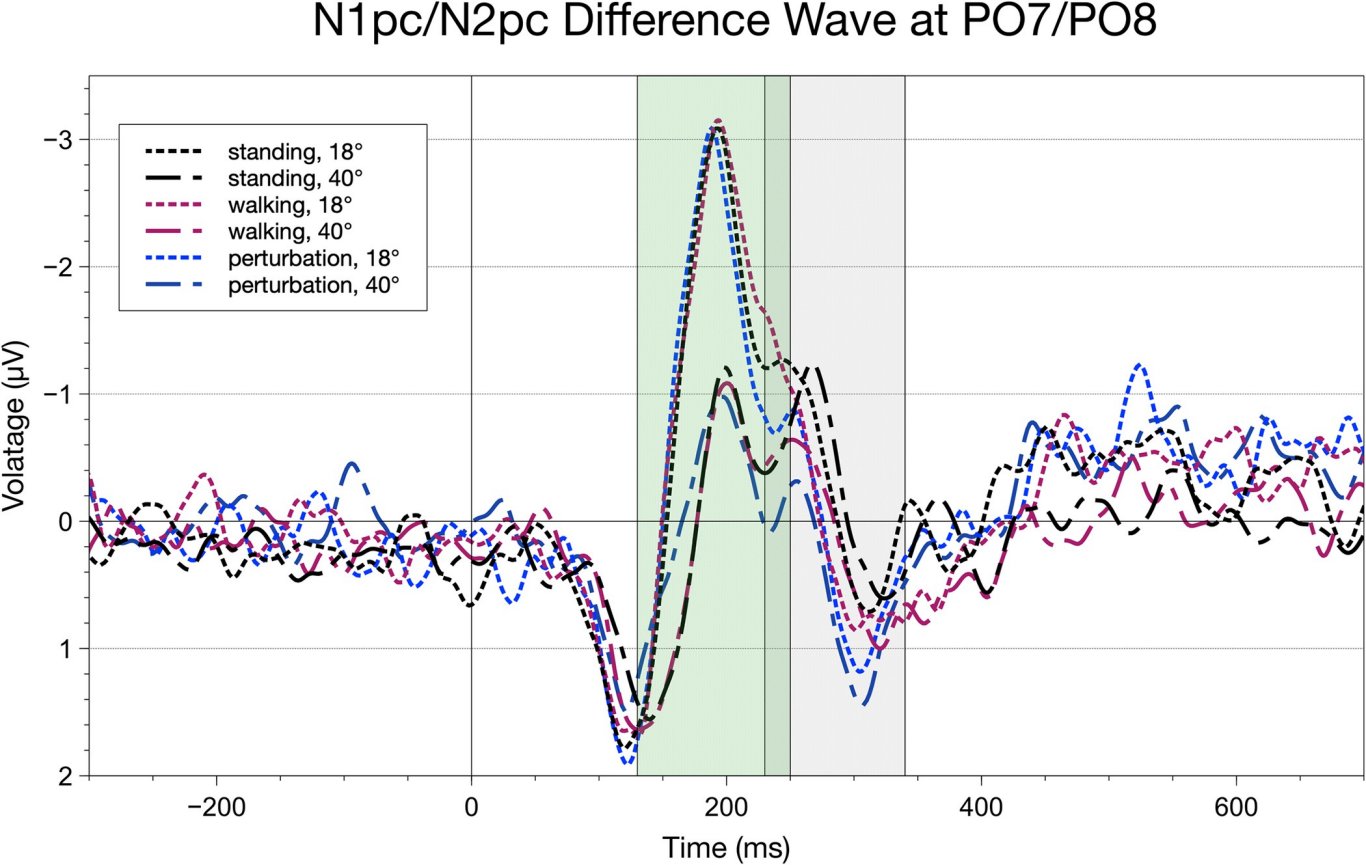
Difference waveforms for all conditions calculated for the ipsi-contra grand average at electrodes PO7/PO8. The green rectangle indicates the fractional area amplitude/latency window to parameterize the N1pc component, the grey one highlights the parameterization of the N2pc component. For the N1pc, significant fractional area amplitude a main effect was found for stimulus eccentricity. N1pc fractional area latencies only indicated an effect of movement complexity. N2pc amplitudes and latencies differed significantly regarding movement complexity.

The analysis of the lateralized ERP components yielded new insights into stimulus processing in a cognitive-motor interference paradigm. By looking into lateralized stimulus processing, we found a stable N1pc/N2pc complex in all possible conditions. Regarding AA, the earlier deflection (first peak of the N1pc/N2pc complex) indicated a main effect only for stimulus eccentricity, F(2,40) = 47.38, p_GG_ < .001, p_crit_ = .05, $\eta_{part}^{2}$ = 0.69, with decreased amplitudes in peripheral (M = 20.36, SEM = 14.90) as compared to extrafoveal stimuli (M = 83.00, SEM = 20.24, t(20) = 6.88, p < .001, p_crit_ = .05, $\eta_{part}^{2}$ = .69). Considering N1pc latencies, there was a movement complexity effect, F(2,40) = 4.37, p_GG_ < .001, p_crit_ = .05, $\eta_{part}^{2}$ = 0.14, with significantly lower latencies when walking with perturbations (M = 196.50, SEM = 4.11) compared to standing (M = 201.97, SEM = 4.17, t(20) = 2.29, p = .03, p_crit_ = .033, $\eta_{part}^{2}$ = .17), or automatic walking (M = 203.48, SEM = 3.08, t(20) = 2.63, p = .02, p_crit_ = .05, $\eta_{part}^{2}$ = .22). For a depiction of the grand averages, see Fig 9.

When looking at the second deflection (second peak of the N1pc/N2pc complex), we could find a similar pattern of significant variations. We found a main effect of movement regarding AA, F(2,40) = 4.04, p_GG_ = .04, p_crit_ = .05, $\eta_{part}^{2}$ = 0.13; here, post-hoc differences indicated higher AA for standing (M = 22.66, SEM = 9.71) than for perturbed walking(M = 7.41, SEM = 4.94, t(20) = 2.32, p = .03, p_crit_ = .05, $\eta_{part}^{2}$ = .17). Latencies indicated a single significant main effect for movement complexity as well, F(2,40) = 3.56, p_GG_ = .04, p_crit_ = .05, $\eta_{part}^{2}$ = 0.11. Post-hoc tests unfolded a significant difference between standing (M = 263.04, SEM = 3.76) and automatic walking (M = 255.44, SEM = 3.85, t(20) = 2.79, p = .01, p_crit_ = .05, $\eta_{part}^{2}$ = .24). A marginal, but insignificant difference could also be found between standing and perturbed walking (M = 256.25, SEM = 4.01) and automatic walking (M = 78.37, SEM = 19.59, t(20) = 1.94, p = .07, p_crit_ = .033, $\eta_{part}^{2}$ = .12). Interestingly, when looking at the graph, there is a special morphology of the walking condition within extrafoveal stimulus eccentricity which is also evident in latency-related post-hoc differences. Here, regular automatic treadmill walking (M = 252.25, SEM = 2.68) was significantly decreased compared to standing (M = 257.92, SEM = 3.72, t(20) = -3.28, p <. 01, p_crit_ = .04, $\eta_{part}^{2}$ = .32) and perturbed walking (M = 255.09, SEM = 4.14, t(20) = 4.39, p < .001, p_crit_ = .05, $\eta_{part}^{2}$ = .47).

## Discussion

This experiment was conducted to shed light on the topic of peripheral vision during locomotion. For this purpose, participants performed a single-target visual discrimination task with varying degrees of stimulus eccentricity while they either stood still, walked, or walked with sideways sways (perturbations) on a tiltable and swayable dual-belt treadmill. To get a grasp on workload measures in the subjective, behavioral, and electrophysiological domain, we used questionnaires (NASA-TLX), response behavior, and mobile EEG. To be able to understand the underpinnings of visual processing in different locomotive states, the study was conducted in a VR-laboratory setting. Overall, our results showed that the mobile, non-seated setting did not impair the quality of measurement, as was shown many times before [36, 54], e.g. finding its expression in the small number of rejected channels and trials. Also, this approach enabled further insights into the ongoing cognitive processes of moving humans. To account for signal noise due to subject movement we used automated correction methods (channel, trial, and IC-rejection) that have been found to reliably produce good signal quality, even in outdoor situations [31, 36].

Concerning the manipulation of movement complexity, we could find a differentiated pattern of results. Regarding the reported subjective workload of the participants, we found that increasing movement complexity did not influence perceived workload considering the unweighted mean of all NASA-TLX subdimensions, contrary to our hypotheses. Still, we could find a significant difference in the physical load subdimension, highlighting that perturbed walking was experienced to impose higher demands than standing or regular walking. This finding acts as a manipulation check regarding the motor conditions as it highlights that sideways perturbations increase the subjectively experienced complexity of movement significantly.

Looking at the results obtained from response times and accuracy, we found additional variables to be significantly altered by our manipulations. Here, we could also include the factor of stimulus eccentricity. Response times varied significantly with motor complexity conditions. Here, we see automatic walking–like the regular walking on a treadmill with fixed speed–did not impose a higher cognitive-motor dual-task load than standing on a spot while performing a rather easy cognitive task in a laboratory environment which is in line with other recent findings [13, 21, 22].

While behavioral results replicated previous findings, neurophysiological measures helped with identifying underlying processes that were not as pronounced in the subjective and behavioral data. We investigated components that are related to inhibitory (frontal N2) and resource-allocation processes (frontal Theta, parietal P3) as well as early, lateralized visual processing (N1pc/N2pc complex). Looking into the movement-related modulation of N2 amplitudes, we found a significantly decreased N2 for regular walking when compared to standing still and perturbed walking. In previous literature, an elevated N2 amplitude was linked to an increased need for inhibition or executive processes [37, 55] with underlying pre-frontal cortical structures being involved. Transferred to this study, walking regularly therefore may have required participants to allocate less attentional resources, rendering the CMI task less demanding. Theta power, on the other hand, was not as informative as the already described ERP measures. Only the Theta power amplitude in the perturbed walking condition differed from standing and regular walking, a result pattern that is most certainly attributable to the balancing reaction to the perturbation itself. A theta increase was often found either in fronto-central [56, 57] or motor areas [58] when the balance was challenged in a comparable way to our perturbations. Regarding parietal P3 measures, amplitudes, and latencies while standing were significantly increased in comparison to both walking conditions. When interpreting P3 amplitudes and latencies in the sense of cognitive resource availability under CMI demands [21, 42], one can see that during standing participants had more resources at their disposal but took longer to process incoming stimuli.

Lateralized visual ERP components indicated a two-staged process of information processing during cognitive-motor interference. The first peak’s latencies of the N1pc/N2pc complex only indicated an influence of movement complexity with perturbed walking having significantly lower latencies than standing and regular walking while amplitudes were not affected by movement complexity. This could be due to the elevated CMI demands regarding the fixed timing of perturbations that appeared at the same point in time in every trial during the perturbed walking condition. Therefore, general arousal was elevated for a short time, giving way for faster cognitive computations of early visual processing. This beneficial impact of perturbation is not evident in later processing stages, but it might give weight to the argument that supraspinal, cortical areas are already involved in very early sections of concurrent cognitive and motor activity.

Regarding the adjunct second peak of the N1pc/N2pc complex, movement complexity had an impact on both the component amplitude and latency. While there was a significant decrease in amplitude for perturbed walking compared to standing still, we found a significantly lower latency for regular walking compared to standing. While the N2pc component which is morphologically closely related to this second peak of our complex is regularly found in paradigms dealing with the visual search of a target within an array of distractors being tightly interwoven with attentional orienting [46, 47, 59], here it seems to be a manifestation of re-entrant processing of stimuli that have a diminished saliency [44, 60]. Movement had an impact on the speed (latency) and intensity (amplitude) of this early visual process. High movement complexity (perturbed walking) seems to diminish attentional resources available to process information efficiently while moderate movement complexity (automatic walking) seems to increase the speed of information processing. Though, when considering post-hoc differences, this effect of faster re-entrant computation only appears when being presented with extrafoveal stimuli. This finding indicates that automatic walking patterns as found on a fixed speed treadmill might help with the detection of and attentional allocation towards visual information in extrafoveal regions of the visual field. These results complement earlier findings that showed that neuronal activity in visual sensory areas is positively influenced by locomotion when it comes to the perception of peripheral visual information [3, 4]. This corresponds to the pattern of the results found for the N1pc/N2pc complex. Here, we can see that the two stages of processing mentioned earlier, namely the stimulus detection and the consecutive deployment of attention, are influenced by locomotive states. As already discussed by Cao & Händel [4], the evaluation of visual stimuli seems to benefit from locomotion. This might be due to an elevated level of arousal or a more specific expansion of the useful field of view. Also when comparing our findings to those of Chen et al. [48] we found differentiation in N1pc/N2pc amplitudes. While they indicated no differences in a selective attention task during standing and free walking, we found automatic walking to influence the re-iterative processing of a lateralized stimulus. This highlights the importance of considering the “automaticity” of a walking pattern when researching cognition in locomotive states.

Stimulus eccentricity manipulations also revealed interesting patterns of results. While we could not investigate the effect of eccentricity on subjective measures (as this was only measured block-wise), we found a response time decrement. When target stimuli were presented at peripheral visual field angles as compared to foveal and extrafoveal areas, response times increased significantly. The same goes for the eccentricity effect on response accuracy where we saw a tendency to miss responses when being presented with peripheral stimuli [2]. Electrophysiological results also showed similar patterns. N2 amplitudes highlighted (marginally) significant decreases from both foveal and extrafoveal stimuli to peripheral ones.

Regarding stimulus eccentricities, amplitudes were decreased significantly only for peripheral stimuli compared to both foveal and extrafoveal ones, whereas latencies were increased. This depicts the relative difficulty of identifying peripheral stimuli, as P3 amplitudes and latencies were also found to index stimulus updating and categorization [61, 62]. With high latencies and low amplitude, this finding might point to the difficulty of updating relevant information when it is coming from a far point in the visual field. Also, the first peak’s amplitudes of the N1pc/N2pc complex that were found to be strongly related to stimulus saliency [44, 60, 61], were significantly decreased for stimuli being presented in peripheral as compared to extrafoveal areas of the visual field. This shows that visual processing was impeded due to the physiological difficulty to correctly perceive and process stimuli of high eccentricity. The second, lateralized peak was not changed by this manipulation, though.

Most interestingly, parietal P3 latencies were sensitive to the combination of both experimental manipulations. These latency interaction effects showed that walking and perturbed walking had lower latencies than standing for foveal and extrafoveal stimuli. Only for peripheral stimuli, mean latencies approached each other, though still differing significantly. This pattern could be due to the increasing general attentional demand that it took to correctly identify the stimulus when stimulus eccentricity was high caused by the decreasing stimulus saliency.

## Limitations

As with every line of research, certain limitations come with our experimental approach. On the one hand, perturbations always occurred simultaneously to stimulus presentation. This ensured that participants were experiencing the highest concurrent cognitive-motor demand right at stimulus presentation, but also gave them the possibility to prepare for the perturbation as there was a cue presentation with a fixed time interval right before. The used movement complexity manipulations aimed at inducing more automatic walking schemes by walking regularly on a treadmill (as comparable to walking on an empty sidewalk for example) and more controlled walking schemes by introducing perturbations (as comparable to walking on uneven terrain). Still, more research is needed in real-world environments to evaluate whether our findings can hold up when moving out of the laboratory environment.

With the simultaneous presentation of stimuli and treadmill perturbation, there is also the possibility that not only the locomotor load solely influenced neurophysiological findings, but also the multisensory inputs going hand in hand with the perturbation motion–auditory mechanical noises and an impact on the somatosensory system. Therefore, future research should take these caveats into account and de-couple external perturbation from experimental stimulus presentation to be able to deduct the underpinnings of cognitive-motor interference processes in a more specific and controlled manner.

Also, like in most mobile neurophysiological studies, motion artifacts might have affected signal quality, though the trial count was very high per condition and averaging increased signal-to-noise ratio drastically. The rationale behind this assumption is that motion artifacts should be distributed as stochastic noise and should not be synchronized to stages of stimulus processing due to induced inter-stimulus interval jitter. To our knowledge lateralized, visual ERP components haven’t been under investigation in mobile settings, therefore the topic needs more research.

## Conclusion

In a single-stimulus visual detection paradigm, the impact of increasing movement complexity (in the form of locomotion) was investigated using questionnaires, behavioral responses, and EEG recordings. Especially EEG helped to understand the cognitive underpinnings of CMI while processing stimuli of increasing eccentricity. Here, we found that foveal and extrafoveal stimuli can be processed efficiently when standing and walking regularly. EEG results indicate that visual stimulus processing might be even more efficient during locomotion, may it be due to an overall increase in arousal or an increase in visual acuity towards more peripheral areas of the visual field. This advantage is lost, though, when visual information is presented outside of the extrafoveal area of the visual field. Returning to the introductory examples, these findings indicate that CMI might not compromise working capacity when stimuli (walking directions, order number, etc.) are presented within a certain degree visual angle and are easy to process. Locomotion might even help with processing task-based visual information if walking is unimpaired by the surrounding environment.
